# 

**Published:** 1821-11

**Authors:** 


THE LONDON
### Medical and Physical Journal.

5 OF VOL. XLVI.] NOVEMBER, 1821. [NO. 273.

### NOTICE.

*Another of the series of* PROEMIA *to the several volumes of this Journal, (which commenced with that to the forty-third volume,) comprising a History of the Progress of Medicine and its auxiliary Sciences for the half-year immediately previous to the period of their production, respectively, was published on the last day of July. One of the especial intentions of those* Proemia, *is to present a comprehensive view of the state and progress of Medicine throughout Europe generally, and in the United States of America; an object that cannot be effected in the regular monthly Numbers of the Journal, because of the small extent of space which can be there appropriated to this purpose.*

### MONTHLY CATALOGUE OF MEDICAL BOOKS.

The London Dissector; or, System of Dissections practised in the Hospitals and Lecture-rooms of the Metropolis: comprising a Description of the Muscles, Vessels, Nerves, and Viscera, of the Human Body; with Directions for their Demonstration. Sixth edition.

Clarke on the Diseases of Females. Part II.

A Treatise on Acupuncturation; being a Description of a Surgical Operation, originally peculiar to the Japanese and Chinese, and by them denominated Zin-King, now introduced into European Practice; with Directions for its Performance, and Cases illustrating its Success. By J. M. Churchill.

Observations on those Diseases of Females which are attended by Discharges. By Charles Mansfield Clarke, Member of the Royal College of Surgeons, and Lecturer on Midwifery in London. Part II. 15s. boards.

[HIGHLEY, FLEET-STREET.]

---

### NOTICES TO CORRESPONDENTS.

*The case of traumatic tetanus, from Quebec, has been received, and will be inserted in the next Number. We beg* Messrs. MERCIER *and* PARANT *to accept our thanks for it.*

*The important papers communicated to us from* Dr. RASORI *will receive an early and due consideration. They will afford another of the numerous illustrations of the quackery of a continental physician, who has dubbed himself* "*chef de* l'Ecole Physio-Pathologique."

Dr. H. *must really pardon us; but we cannot, and are determined not to admit statements of cures in cases that are said to have proved rebels to the regular administration of remedial agents by other practitioners, unless the mode of treatment which is asserted to have proved successful at last, be detailed at the same time. He verges on the borders of quackery who pursues a different course.*

*Numerous books have reached us for review. We will endeavour to introduce them to the acquaintance of our readers in regular succession. No other distinction shall be made as to precedency, except between intrinsic worth and a mere gaudy exterior,—between solid materials and the tinselled ornaments that shine with a false lustre.*

*What care we for the wrath of* LONDINENSIS? *As long as we conscientiously discharge our duty, and study to merit the approbation of the good, we need not feel hurt at the obloquy of the few.* LONDINENSIS *has taken care to add* M.C.S. *to his assumed appellation. We think he might, with more propriety, have suppressed the two first letters, and substituted the conjunction* AS *in their place. There would then have been a more natural connexion between his honorary initials and his real name.*

H. L. *need not be at the trouble of disguising his hand. We venture to predict that, through life, he will never be otherwise than* a-nonymous.

*We give full credit to* Mr. WALLIS *for his anxiety to establish a proper discriminating mark in cases requiring venous depletion; but we must decline admitting a* suppositious *case in illustration of his doctrine. We shall be happy to receive something more practical from that gentleman.*

*Our attention has been earnestly called to the shameful practice of certain* pedlar-*vaccinators, who, with an empty purse in one hand, and a blazoned diploma, on which the Jennerian name is shamefully prostituted, in the other, wander about the country, and impose on the credulity of the humane and the charitable. We had hoped that the very able manner in which these vaccine swindlers had been exposed by* Mr. RING, *would have rendered any further notice of them, on our part, unnecessary. We have, however,*

*been lately put in possession of documents, which, unless these abuses be speedily reformed, we shall deem it our duty to lay before the authorities delegated to watch over the public health, in order that proper measures may be adopted to put an end to so disgraceful a traffic. We beg to state, by way of a gentle hint to the promoters of bastard Institutions for the pretended propagation of vaccine, to whom the majority of failures seem due, that we have taken a legal opinion on the subject, and that we find all such* pedlar-vaccinators *to come within the scope of the "Vagrant-Act." The very respectable practitioner at Chelmsford, who has furnished us with the information upon which we have descanted, is entitled to the most grateful acknowledgments of the profession and the public.*

*If* "a Pupil to a Lecturer on Midwifery" *can authenticate his statement, we pledge ourselves to lay it before the public.*

Dr. PARKEMAN's *communication from New-York has not been noticed before, through mere inadvertence. That gentleman informs us, that the trade of slander among the medical profession in America thrives with as much success as in the Old World. In a late trial before the supreme judicial court at New-York, a Dr. Niles obtained a verdict of damages, to the amount of* 600 *dollars, as a compensation for having been called a "Sangrado licentiate."*

*We have received the pamphlet intitled* "Thoughts on the Establishment of Public Baths." *We fully agree with the writer as to the importance of the measure he proposes; and we trust that his project will, at no very distant period, be carried into execution. Something, however, in a more tangible shape than a mere address should be brought forward; and we will cheerfully give it whatever support may be derived from the recommendation of the Medical and Physical Journal.*

*We regret much that* Mr. SMERDON's *valuable paper reached us after the department for Original Communications was completely filled. It shall appear in our next without fail.*

*The same apology must serve us with* Mr. YATEMAN, *whose reply to* "an Old Practitioner" *shall certainly appear in our next.*

*A* "CONSTANT READER," *from Manchester, is desirous of knowing whether any of his* medical *brethren have met with the very extraordinary occurrence of "people about the middle period of life" losing their hair with scurfy desquamation, and without any known cause. Our Correspondent does not go the length of puffing the Russia oil in such cases; but it is evident that he would have done so, had he either been* bald *enough, or we less* long-pated. *We trust his next communication will bear the stamp of "paid" upon it.*

Mr. B. HUTCHINSON, *whose case of tic douloureux, cured by carbonate of iron, was already printed and inserted in the first part of the present Number, when his letter of the 23d ultimo reached us, is anxious that we should announce that the patient "has since suffered from a trifling relapse." This fresh mark of candour is peculiarly creditable to* Mr. B. HUTCHINSON.

*We are anxious to express our warmest acknowledgments to our Madrid Correspondent, for the readiness with which he has transmitted to us the first volume of the* Decadas Medico Quirurgicas. *We shall not fail to make extracts from it in our next Numbers.*

Dr. HANNAY's *communication, from Liverpool, arrived too late for the present Number.*

